# Moving beyond the lines: lung ultrasound pixel-wise computer-assisted analysis for critically ill patients

**DOI:** 10.1186/s13054-022-04219-2

**Published:** 2023-02-22

**Authors:** Orphée Faucoz, Denis Standarovski, Amazigh Aguersif, Sihem Bouharaoua, Benjamine Sarton, Stein Silva, The MAGELLAN Study Group

**Affiliations:** 1 French National Center for Spatial Studies (CNES), Calculation and Data Engineering Department, Toulouse, France; 2 Critical Care Unit, University Teaching Hospital of Purpan, Place du Dr Baylac, 31059 Toulouse Cedex 9, France; 3 UMR INSERM/UPS 1214, UPS, Toulouse NeuroImaging Center, Toulouse University, 31059 Toulouse Cedex 3, France

Dear Editor,

Lung ultrasonography (LUS) has become an essential component of the evaluation and clinical management of patients admitted to the intensive care unit (ICU). The interpretation of LUS artifact (A- and B-patterns), analysis of the pleura, and the visualization of real images (C pattern) have demonstrated usefulness for the differential diagnosis of acute respiratory failure (ARF) [1]. However, current methods are non-quantitative and have important drawbacks deriving from visually guided assessment of LUS data [2]. Interestingly, recent in vitro and in vivo studies suggest that LUS data carry valuable information that correlates with lung density [2]. This led to the hypothesis that the loss of lung aeration can be quantified on the basis of the type, the number and the extent of LUS patterns detection. Many scoring systems have been proposed to achieve this important goal [1]. Nevertheless, it is commonly acknowledged that these methods, which are based only on medical experts’ analysis, can be time-consuming, are user-dependent, and hold the risk of leading to oversimplified and potentially harmful diagnosis algorithms, particularly in the complex pathophysiological setting of critically ill patients.

To accurately cope with these issues, computer vision approaches, built upon machine learning (ML) algorithms have been recently proposed. These methods have potential to provide new computer-aided diagnosis for LUS data that could transform the way in which ICU practitioners assess and manage critically ill patients. However, it is worth noting that the data available in this field have demonstrated variable classifier’s accuracy and were exclusively limited to the analysis of B-lines [2].

Aiming to lay the foundation needed to develop an end-to-end tool for computer-assisted analysis of critically ill patient’s LUS data, we followed a knowledge transfer approach from satellite to medical imaging based on semantic segmentation and signal processing. Semantic segmentation is a form of pixel-level prediction where each pixel in an image is classified according to a category using deep learning methods. In order to do this, we designed a proof-of-concept study built upon one of the largest LUS datasets from severe COVID-19 patients reported to date. Hence, we prospectively recruited adult COVID-19 patients who were in ARF (defined by blood oxygen saturation as measured by pulse oximetry < 90% while breathing room air or respiratory rate > or = 30 breaths/min) at hospital admission (University Hospital of Toulouse and Cayenne Hospital, France, between July 2020 and March 2021). Exclusion criteria were patient’s history of chronic respiratory disease and the lack of LUS image. The study was approved by the ethics committee of the University Hospital of Toulouse, Toulouse, France (Ref. 2020-A01225-48), and written consent was obtained from all participants. Overall, 5000 LUS frames from 78 patients affected by COVID-19 with different degrees of severity were gathered and labeled (Additional file 1).

Thereby, we provide a new automatic pixel-wise classifier, which was able to accurately identify for the first time, all the main LUS patterns that can be observed in this setting (overall Aires Under the Curve, AUC = 0.97; pleural line AUC = 0.99; A pattern AUC = 0.96; B pattern AUC = 0.97; C pattern AUC = 0.95; please see Fig. 1d–h). Furthermore, because the use of artificial intelligence methods for the development of computer-based aid to medical decision tools can be hindered by the poor explicability of the obtained results, we increased the explicability of our LUS data classifier, by using machine learning methods not as a black box, but as a pre-processing step to a signal analysis process that refine the result of the network. Doing so, our numerical solution was able to provide real-time meaningful feedback to physicians at patient’s bedside by highlighting the areas of the image that were most contributory to the model’s classification decision (Fig. 1a–c).

**Fig. 1:**
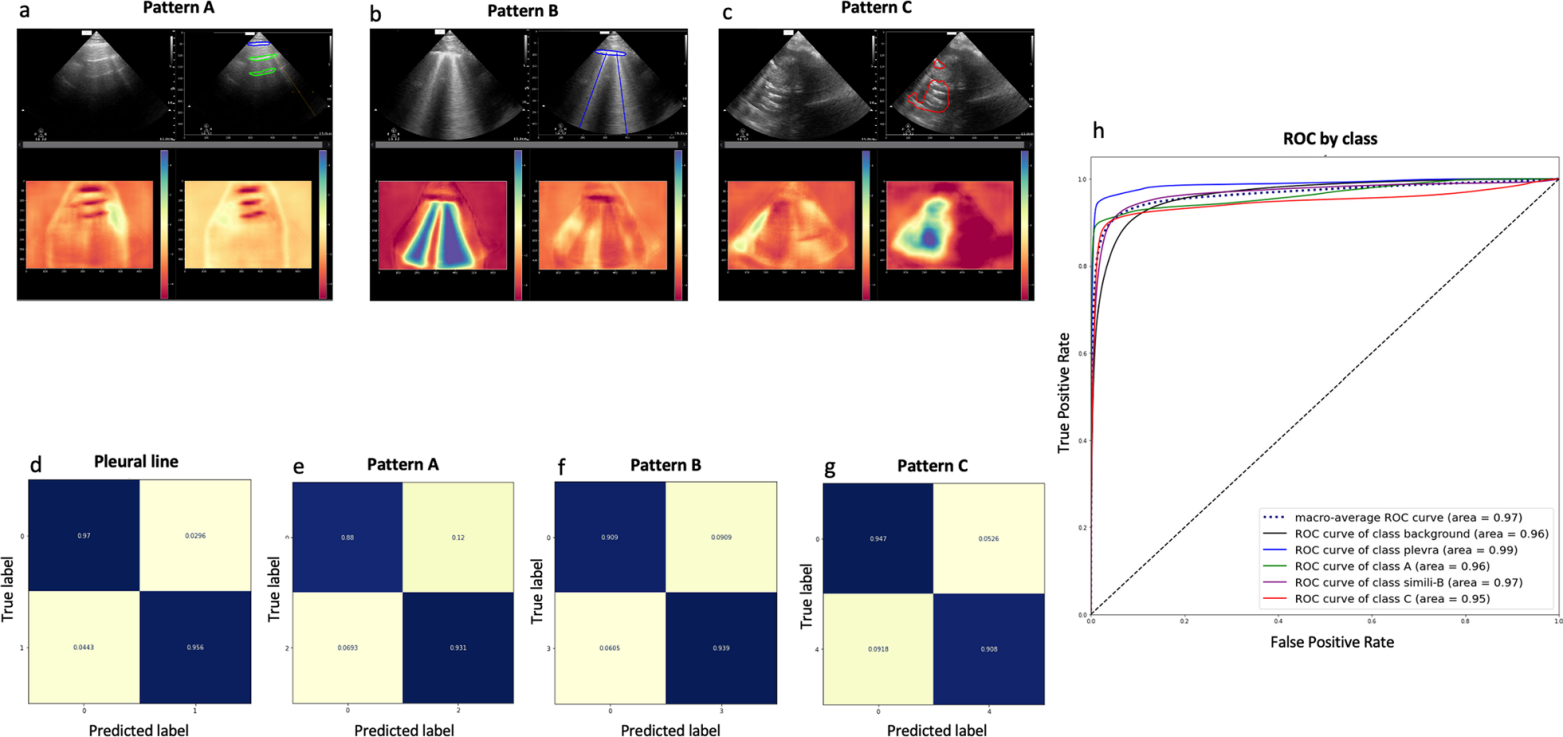
Automatic pixel-wise LUS image classification based on semantic segmentation. LUS patterns are described following current guidelines [1]: A: normal; B: lung edema; C: alveolar consolidation; pleura: pleural line detection (please see Additional file 1 for additional information). **a**–**c** Four-panel views to allow the estimation of the perceptual quality of the fully automated classifier for each LUS pattern. Each 4-panel represents both the neural network input (right upper panel: raw LUS image; left upper panel: annotated LUS images) and output (right and left lower panels: heatmaps highlighting the area of the image that were most contributory to the model’s classification decision). **e**–**g** Confusion matrix computed for each LUS pattern (pleural line, 1-**d**; pattern A, 1-**e**; pattern B, 1-**f**; pattern C, 1-**g**) on a voxel-wise level. **h** The LUS final segmentation was very accurate (F1 = 0.966, Recall = 0.966; Precision = 0.966). Aires under the curve (AUC) values are depicted for of each LUS patterns detection

We think that our work opens the door toward plausible improved diagnosis [1], automated severity scoring [2], medical triage [3], monitoring [4] and personalized treatment [5] for critically ill patients with acute respiratory failure. Overall, the eventual integration of this model into ultrasound hardware seems plausible as a method to ‘move beyond the lines’ and ultimately improve outcomes through a combined use of LUS with other components of critical care ultrasonography [1] to yield a more holistic, comprehensive and accurate evaluation of the critically ill patient at point of care.

## Supplementary Information

**Additional file 1**. For additional information regarding artificial intelligence derived methods.

## Data Availability

The data that support the findings of this study are available on request from the corresponding author. Computer codes will be shared using a permanent and public repository that issues citable digital objects identifiers (GitHub).
