# Supplementary material for: Moving beyond the lines: lung ultrasound pixel-wise computer-assisted analysis for critically ill patients
Source: Crit Care. 2023 Feb 22;27:68. doi: 10.1186/s13054-022-04219-2 (PMC9944795; doi:10.1186/s13054-022-04219-2)
Supplement: Supplementary file 1 — Additional file 1. For additional information regarding artificial intelligence derived methods. [file 13054_2022_4219_MOESM1_ESM.docx]

**Supplementary Material**

**Materials and Methods**

*Study design*

The whole dataset, was prospectively collected at the ICUs from both the University Hospital Purpan (Toulouse, France) and Cayenne Hospital (French Guyana, France) between July 2020 and March 2021. Patients were managed by physicians according to current guidelines and recommendations for critically ill COVID-19 patients. The study was approved by the ethics committee of the University Hospital of Toulouse, Toulouse, France (“Comité Consultatif pour la Protection des Personnes”, Ref. 2020-A01225-48); written consent was obtained from all participants. COVID-19 diagnoses were confirmed by positive real-time reverse transcription polymerase-chain-reaction (RT-PCR) assay for pharyngeal swap specimens.

*Population*

We prospectively recruited adult COVID-19 patients who were in acute respiratory failure (ARF) at hospital admission. ARF was defined as patient’s blood oxygen saturation as measured by pulse oximetry < 90% while breathing room air or respiratory rate > or = 30 breaths / min. Exclusion criteria were patient’s history of chronic respiratory disease and the lack of LUS image.

*Lung ultrasound examination*

All patients underwent a LUS assessment by senior critical care practitioners, with advanced level of thoracic ultrasound training (AA, SB, SS). The level of agreement between raters for the LUS findings has been previously reported. Lung ultrasound assessment was performed with HP Sonos 5500 (Hewlett-Packard Development Company, LP) and Sonosite M-Turbo (Fujifilm Sonosite Inc, WA, USA) 2- to 4- MHz probes. As previously reported, six quadrants were defined for each hemithorax. Following international guidelines for LUS study and data reporting, we used consensual semiotics criteria. The normal pleural line was defined as a horizontal hyperechoic line visible below the rib line. Pleural effusion was defined as a hypoechoic collection limited by the diaphragm and the pleura (PE profile). A normal lung pattern was defined as the presence in a quadrant of lung sliding with reverberating horizontal A lines (A profile). Alveolar consolidation was defined as the presence of poorly defined heterogenous wedge-shaped hypoechoic images. We distinguished two patterns of alveolar consolidation during COVID-19: subpleural non-translobar (C1 profile) which might correspond to peripheral lung embolism, and posterior translobar with occasional mobile air bronchograms (C2 profile). Alveolar-interstitial syndrome was defined as the presence of more than two vertical lines B lines in a given lung region. To specifically address the usefulness of LUS evaluation to provide semi-quantitative pulmonary oedema assessment, we defined three B-lines classes: B1 profile (thin, multiple and well-defined), B2 profile (large and coalescent), B3 profile (“shining white lung”).


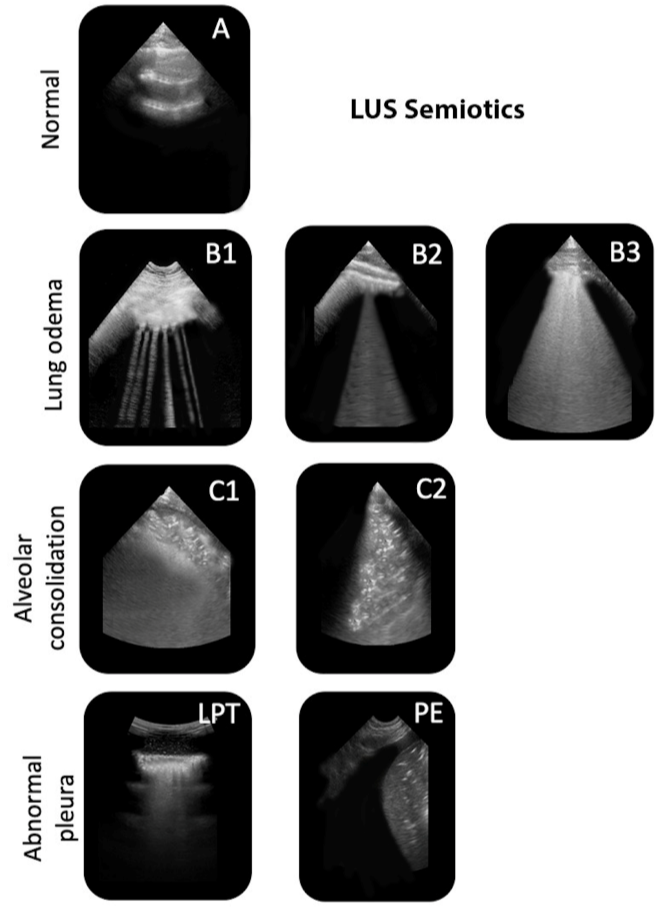


*Data annotations*

LUS frames were annotated by 3 medical experts (AA, SB, SS) from Critical Care Units of University Hospital of Toulouse (Toulouse, France) and Hospital of Cayenne (French Guyana, France). These raters are all senior critical care practitioners, with advanced level of thoracic ultrasound training. LUS frames from patients affected by COVID-19 with different degree of severity were gathered and labeled using Clickpoints© annotation tool. Ten random frames of each scan are used to ensure all patterns can be seen yet avoiding too much data redundancy. In addition to LUS labels, we also used as input annotated pleural line, rib, diaphragm, liver and spleen images. It is worth noting that in order to extract all relevant information from our LUS dataset, most of the not noisy frame pixels have been labeled, making it possible for the network and post-processing to get additional features in the analysis.

*Pre-processing*

In order to lower the bridge between the different ultrasound systems, data augmentation was used. Indeed, artificially increasing LUS images by mixing the contrast, gamma and added blur helped move one image from one ultrasound system modality to another. As some patterns features are close, similar classes were merged into one super-class (e.g., B superclass). In addition, minor classes not helping in the segmentation process were discarded (e.g., spleen). Eventually, the dataset used in the training process is composed of 5 classes: Pleural Line, A pattern, B superclass, C pattern and background.

*LUS patterns segmentation*

Semantic segmentation is the process of classifying each pixel of an image. In the current use case, a semantic segmentation neural network is used to emphasize the disease markers. Those markers were present and annotated in the dataset. The function Φ of the network maps an input LUS image X to a multi-class Z image with equal dimensions, so that every pixel of the i class of Z is detected as a class pixel or non class pixels.

In most semantic segmentation network, Φ is divided in two stages : Φenc learns the features inside the image and Φdec transpose the learned features into a x × y × i Z segmentation output.


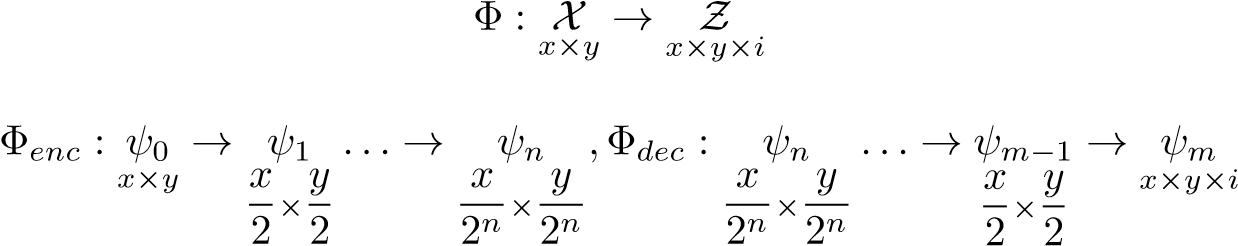
Both Φenc and Φdec are composed of convolution and activation layers ψ learned during the training process. As Φenc reduce the dimension as the layer continue, Φdev increase layers dimensions. The final ψm layer of Φdec is a x × y × i matrix where every pixel is assigned to a class probability :

It is important to pick a correct loss for accurately training the network. Regular training of segmentation network uses the cross-entropy (CE) loss. However, in unbalanced class distribution settings, CE will be biased toward the majority class. In LUS images, most pixel belongs to the background class, training a network with CE will result in blurry frontier between relevant information and background. To counter balance this bias, our network is trained with a composition of two losses, focal loss L*_FL_* and boundary loss L*_BL_*.


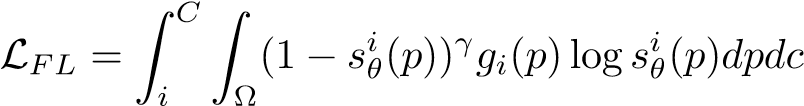
 (3)

| Z  L*_BL_* = ∆*_G_*(*p*)*s_θ_*(*p*)*dp* | (4) |
| --- | --- |

Ω

L = L*_FL_* + *α*L*_BL_* (5)

Where sθ is the last layer ψm of Φdec, g is the ground truth, p ⊂ Ω, the set of pixels, ∆G is a function returning −Dp or Dp with Dp being the L2 distance from p to the pattern contour of gi, α is a constant to balance the two losses. As seen in Eq. 3, LFL weights hard to classify example. This is essential for segmenting C-patterns that are rare and have difficult features to learn. LBL adds region-wise distance which is helpful to have strong segmentation boundary, it forces strong gradient from pattern region to background. Several network architectures have been tested: U-Net, DeepLab-v3, LinkNet, MANet and YNet.


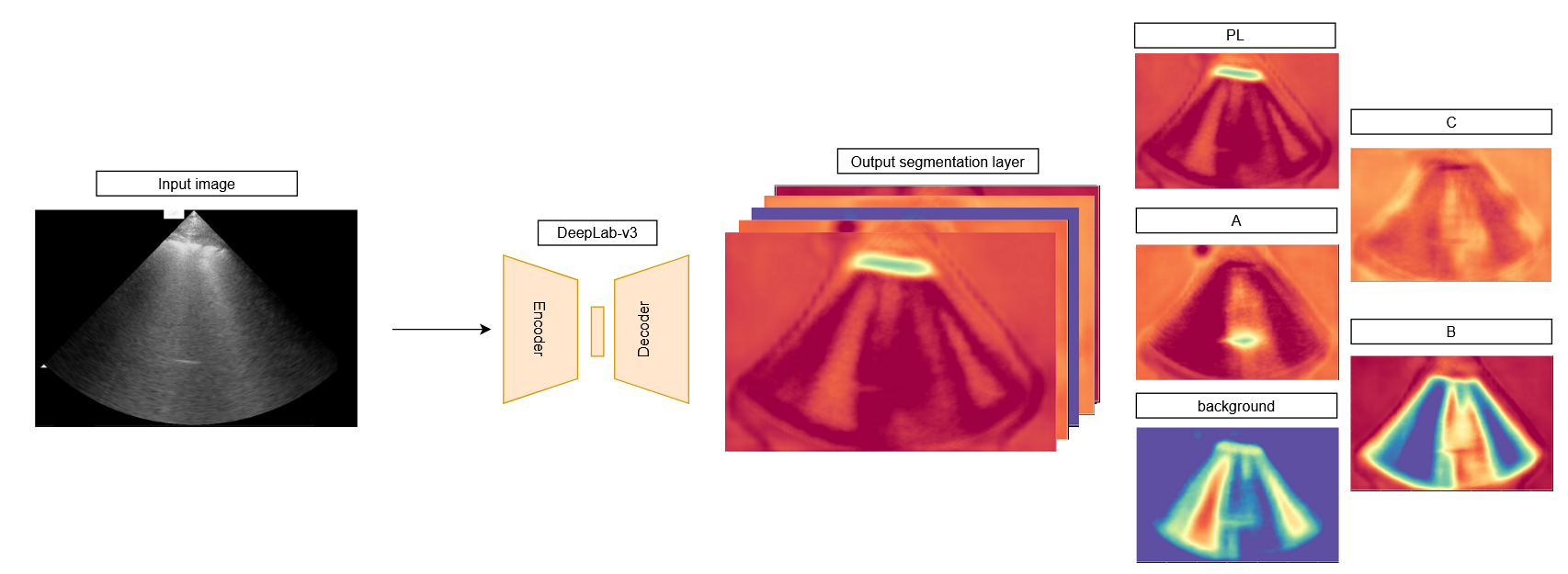


*Metrics*

To assess the performance of the different architectures the F1 metric was chosen. F1 is the harmonic mean of precision and sensitivity. This follows the use of focal and boundary losses. Precision highlights well classified pixels that are not outside of patterns region whereas recall shows if all pixel belonging to patterns are found. For regions where we want near perfect delimitation, such as B, PL and A, precision will be an important metric. C-pattern is harder to classify and the pattern region is uncertain, in this case it is preferred to force the detection, networks showing high recall will be favored. Additionally, ROC curve (receiver operating characteristic curve) displaying the relation of true positive rate against false positive rate at different classification thresholds, was also computed.

*Post processing*

The final layer *ψ_m_* of the semantic segmentation network (Eq. 2) contains the pixel-wise classification for the 5 classes. This view can be directly used as a processed view of a LUS image. However, some information is lacking, it does not give an immediate overview of the lung condition of a patient. Ideally, a post-processing analysis on top of *ψ_m_* would give a counting of B-Lines with their dimensions and a discrimination of the sub-classes of B superclass.

The B superclass was initially composed of 4 classes: B well defined, B large, White lung and Z-Line. Those sub-classes were particularly hard to discriminate one from another. Indeed, drawing the frontier from a B2 to a B3 is difficult as well as classifying a well-defined B-Line not erasing the A-Line as a Z-Line. This analysis may differ depending on the physician view. To tackle this problem, we introduce a post-processing tool based on signal analysis. A LUS image can be seen as a cone starting from an origin point O(*x_o_,y_o_*) where the first angle *θ*_0_ would be defined by the leftmost line from O to the furthest left point T*_l_* and the final angle *θ_r_* would be defined by the rightmost line from O to the furthest right point T*_r_* of the LUS image. {O, T*_l_*, T*_r_* } defines a triangle containing all the angles of the LUS image. From this triangle each line from each angle can be computed using the Bresenham’s algorithm. For every two points O(*x_o_,y_o_*) and P(*x_p_,y_p_*) in X , the Bresenham’s algorithm(BA) gives a list of pixels *L_i_* crossing the line.

*BA*(XO*,*T*l,*T*r*) = *Ll*→*t* (6)

The *L* grid can be mapped directly on *ψ_m_* to retrieve class *i* signature alongside each *θ* angle.

*Lθ*(*ψm,i*) = *ωθ,i* (7)

*ω_θl_*→*_θr,B_* will then be the distribution of B pattern around the cone of the LUS image. Pixels not belonging to the *B* class can be filtered out by applying a simple threshold on the class probability. From *ω_θ,B_* a distribution *µ_θ,B_* of the number of points belonging to the *B* class on angle *θ* is obtained. The *µ_θl_*→*_θr,B_* distribution is enhanced with a 1-D Gaussian filter *G_B_* removing negligible local variations. ∆*_grad_* is the gradient linked to the *G_B_*(*θ*) function.


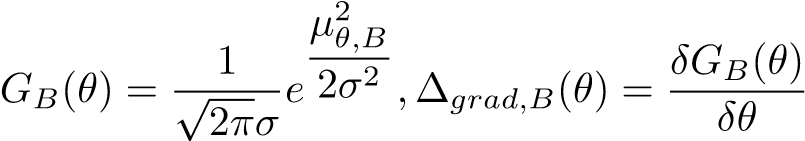


From GB(θ) and ∆grad,B(θ), local maxima are picked, they are the sources of B-Lines. By walking on the gradient and comparing ∆grad,B(θsource) with ∆grad,B(θθsource → θl) and ∆grad,B(θθsource → θr), the B-Line dimension can be found. If the gradient is inverted, then another B-Line is starting, if the gradient is flattened, this marks a limit of the current B-Line.

With B-Line source θsource and dimensions {θlb,θrb}, the B-Line subclass can be discriminated. A White Lung would not find neighbors during the ∆grad,B(θθsource → θl) and ∆grad,B(θθsource → θr) walks. B-Lines θsource stopped crossing A-Lines determined by ψm,A will be detected as Z-Line. Alternatively, θsource starting while ψm,PL could not see PL could be a sign of C-pattern that can be consolidated by ψm,C.

**Results**

*Ultrasound data*

Overall, 5000 LUS frames from 78 patients affected by COVID-19 with different degree of severity were gathered and labeled. Data analysis was conducted on CNES (French National Center of Space Studies) HPC platform, namely on NVIDIA A100 and V100 cards. The total amount of frames was divided 5-fold: 4 folds were used for the training and the last one for validation. The validation fold was created in a fair representation of each pattern. The visual results were conducted using an independent fold on frames which was not initially annotated by the physicians.

| **Labelled** | **Elements** |
| --- | --- |
| Number of patients | 78 |
| Number of scans | 510 |
| Number of labelled frames | 5000 |

| **Annotation pattern summary** | |
| --- | --- |
| Pleural Line | 4214 |
| B-line large | 2627 |
| A-line | 1397 |
| B-line well-defined | 755 |
| C-pattern | 621 |
| No pattern | 586 |
| White Lung | 367 |
| Z-line | 308 |
| Air Bronchogram | 235 |
| Pleural effusion | 228 |
| Rib | 176 |
| Diaphragm | 103 |
| Spleen | 8 |
| Liver | 2 |
| **Total Annotations** | 11627 |

*Segmentation accuracy*

When networks were close one to another, DeepLab showed better result on recall of different patterns and so was chosen as standard segmentation model for the signal analysis. The ROC curve in Figure X was computed using the DeepLab segmentation model. It is worth noting, if PL was almost perfectly segmented, alternatives patterns such as B and C were harder to segment. We suggest that this result might be related to the fact that PL features (position, shape) are close for every frame whereas other patterns are based on more heterogenous ultrasonographic signs. In addition, C-pattern frontier were semantically hard to estimate, probably because this result in the network being sometimes too greedy or restrictive compared to the clinician label.

| Network | F1 | Recall | Precision |
| --- | --- | --- | --- |
| MANet | 0.9662 | 0.9665 | **0.9669** |
| LinkNet | 0.9656 | 0.9656 | 0.9661 |
| DeepLab | **0.9663** | **0.9666** | 0.9662 |
| YNet | 0.9618 | 0.9594 | 0.9649 |

*Explainability of results*

If the segmentation metrics gives relevant information on the validation set, estimating the perceptual quality of the post-processing analysis on the unlabeled test set cannot be done with metrics. The last layer of the segmentation model is normalized to give a heatmap on each pattern; the resulting map is then a highlighted view for the clinician. Hence, as Figure 1 shows, the physician in charge of the patient, might be given a multi-view panel, encompassing images from the input to the post-processing output with both ψm,B and ψm,C that highlights relevant pixels. As the results in Figure 1 shows, the key activation areas for all classes included the pleural line and the main axis of B lines (B pattern).
